# From cytogenomic to epigenomic profiles: monitoring the biologic behavior of *in vitro *cultured human bone marrow mesenchymal stem cells

**DOI:** 10.1186/scrt138

**Published:** 2012-11-20

**Authors:** Serena Redaelli, Angela Bentivegna, Dana Foudah, Mariarosaria Miloso, Juliana Redondo, Gabriele Riva, Simona Baronchelli, Leda Dalprà, Giovanni Tredici

**Affiliations:** 1 Department of Surgery and Interdisciplinary Medicine, University of Milan-Bicocca, via Cadore 48, 20900, Monza, Italy; 2 S. Gerardo Hospital, Medical Genetics Laboratory, via Pergolesi 33, 20900, Monza, Italy

## Abstract

**Introduction:**

Bone marrow mesenchymal stem cells (BM-MSCs) are multipotent cells that can differentiate into different cell lineages and have emerged as a promising tool for cell-targeted therapies and tissue engineering. Their use in a therapeutic context requires large-scale *in vitro *expansion, increasing the probability of genetic and epigenetic instabilities. Some evidence shows that an organized program of replicative senescence is triggered in human BM-MSCs (hBM-MSCs) on prolonged *in vitro *expansion that includes alterations in phenotype, differentiation potential, telomere length, proliferation rates, global gene-expression patterns, and DNA methylation profiles.

**Methods:**

In this study, we monitored the chromosomal status, the biologic behavior, and the senescence state of hBM-MSCs derived from eight healthy donors at different passages during *in vitro *propagation. For a more complete picture, the telomere length was also monitored in five of eight donors, whereas the genomic profile was evaluated in three of eight donors by array-comparative genomic hybridization (array-CGH). Finally, an epigenomic profile was delineated and compared between early and late passages, by pooling DNA of hBM-MSCs from four donors.

**Results:**

Our data indicate that long-term culture severely affects the characteristics of hBM-MSCs. All the observed changes (that is, enlarged morphology, decreased number of cell divisions, random loss of genomic regions, telomere shortening) might be regulated by epigenetic modifications. Gene Ontology analysis revealed that specific biologic processes of hBM-MSCs are affected by variations in DNA methylation from early to late passages.

**Conclusions:**

Because we revealed a significant decrease in DNA methylation levels in hBM-MSCs during long-term culture, it is very important to unravel how these modifications can influence the biologic features of hBM-MSCs to keep track of this organized program and also to clarify the conflicting observations on hBM-MSC malignant transformation in the literature.

## Introduction

Bone marrow mesenchymal stem cells (BM-MSCs) are multipotent cells that can differentiate into different cell lineages [1]. Human BM-MSCs (hBM-MSCs) are easily isolable and are not ethically restricted; thus they have emerged as a promising tool for cell/gene therapy for tissue regeneration and anticancer treatments. Their application is concurrently tested in various clinical trials [2], but their use requires large-scale *in vitro *expansion, increasing the probability of genetic and epigenetic instabilities. Spontaneous transformation of mouse BM-MSCs has been observed [3-6]; chromosomal instability has also been evidenced for rat BM-MSCs [4,7]. Conversely, confounding data exist about the stability of hBM-MSCs and their ability to transform spontaneously *in vitro *[3,5,8-12]. Some authors have reported spontaneous transformation of human MSCs, but in several cases, the data were retracted, because the occurrence of transformed cells was due to cross contamination of the original cell culture with tumor cell lines [13-15].

Although, to date, hBM-MSCs appear to be less prone to malignant transformation during *in vitro *culture, more-detailed studies are urgently needed to evaluate their *in vitro *behavior, particularly as a great variability in terms of proliferative capacity and life span was evidenced between donors [8]. However, hBM-MSCs have a restricted life span *in vitro*, as does any normal somatic cell, because of the phenomenon called the Hayflick limit [16], or replicative senescence, whereby they exhibit a reduced differentiation potential, a shortening of the mean telomere length, and morphologic alterations [17,18]. It is now evident that a strong correlation exists between DNA methylation-stem cell renewal-differentiation, as well as between stem cell culture-copy number changes-spontaneous malignant transformation (see reviews [19,20]). Recent studies on replicative senescence of hBM-MSCs have demonstrated that gene-expression changes are continuously acquired with increasing passages, influencing their differentiation potential [21]. Moreover, DNA methylation-pattern variations in hBM-MSCs have been seen to overlap in long-term cultures and in aging *in vivo*, suggesting that replicative senescence and aging are regulated by specific epigenetic modifications [22].

The purpose of this study was to track the chromosomal status, the biologic behavior, and the senescence state of hBM-MSCs derived from eight healthy donors at different passages during *in vitro *propagation. First, we applied the conventional cytogenetic technique to observe major (>2 Mb) and minor structural abnormalities and to identify low mosaic conditions; subsequently, a more-detailed whole genomic analysis by array-comparative genomic hybridization (a-CGH) was conducted. In addition, the telomere length was monitored to assess cellular aging *in vitro*. Finally, to evaluate DNA methylation-pattern changes after long-term *in vitro *expansion, a genome-wide analysis of DNA methylation was performed comparing early and late passages, and the results were further analyzed by gene ontology (GO) functional analysis.

## Materials and methods

### Isolation, immunophenotyping, and culture of hBM-MSCs assay

hBM-MSCs were obtained from bone marrow in excess from eight anonymous healthy donors undergoing marrow harvests for allogenic transplantation at San Gerardo Hospital (Monza, Italy). Donor ages were between 20 and 45 years. An informed written consent was obtained from all the subjects, according to the national ethical guidelines. Mononuclear cells, obtained after centrifugation of the harvested bone marrow in a Ficoll-Hypaque column, were suspended in Dulbecco Modified Eagle Medium (DMEM; Lonza, Verviers, Belgium) containing 10% fetal bovine serum defined (FBS; Hyclone, Logan, UT, USA), plated in 75 cm^2 ^(T75) culture flasks, and maintained at 37°C in a humidified atmosphere with 5% CO_2_. At this time, cells were considered to be at passage 0 (P0). After 48 hours, the nonadherent cells were removed and the cells attaching to the culture flasks were cultured in DMEM plus 10% FBS defined, 2 m*M *L-glutamine, 100 U/ml penicillin, 100 μg/ml streptomycin, 250 μg/ml fungizone (Lonza), with a change of medium every 3 to 4 days. When cultures reached 80% to 90% of confluence, cells were washed with Dulbecco Phosphate Buffered Saline (PBS; Sigma-Aldrich, St. Louis, MO, USA), detached by using 0.25% trypsin in 0.1% EDTA (Lonza), and re¬plated (1/3) in 75-cm^2 ^culture flasks.

The immunologic characterization of hBM-MSCs was performed with flow-cytometric analysis at the Tettamanti Foundation laboratories (Monza, Italy) by using specific antibodies for the membrane antigens CD33, CD34, CD90, CD105, HLA-DR, and HLA-ABC [23]. Osteogenic, adipogenic, and chondrogenic differentiation of hBM-MSCs was performed by using standard protocols adopted in our laboratory [7,24,25].

### hBM-MSC growth curves

hBM-MSC growth curves were obtained by plating cells on 60-mm-diameter dishes, at a density of 70 to 100 × 10^4 ^cells, and counting cells after 24, 48, 72, and 96 hours from plating. Population-doubling time (PDT) was calculated with the following formula:

PDT = (CT × ln2)/ln (Nf/Ni)

where CT is cell-culture time, Ni is the initial number of cells, and Nf is the final number of cells.

### Senescence β-galactosidase staining assay

The amount of senescent cells was determined in hBM-MSC cultures, from eight healthy donors and at different passages during *in vitro *propagation, by using the Senescence β-Galactosidase Staining Kit (Cell Signaling Technology, Danvers, MA, USA), according to the manufacturer's instructions. Cells were seeded at a density of 1 × 10^4^/cm^2 ^into 24-well plates and cultured for 24 hours before senescence-associated β-galactosidase staining. The SHSY-5Y neuroblastoma cell line was used as negative control of the β-galactosidase staining. At the end of the staining procedure, representative images were taken from diverse areas of each cell culture by using phase-contrast microscopy. For the determination of the senescent cell percentage in each culture, an average value was calculated by counting, in eight random fields, the total number of cells and the number of cells with intracellular blue dye.

### Karyotype analysis of hBM-MSCs

Metaphase chromosome spreads were prepared from 70% to 80% confluent cultures at the designated passage, according to standard procedures. Actively dividing cells were treated with colcemid overnight at 37°C. Cells were combined in 1% sodium citrate:0.56% KCl (1:1) for 20 minutes at 37°C. Cells were fixed with methanol/acetic acid (3:1). Chromosome analysis was carried out by applying Q-bands by fluorescence using quinacrine (QFQ banding), according to routine procedures, following the guidelines of the International System for Chromosome Nomenclature 2009 (ISCN 2009) [26]. On average, 25 metaphases were evaluated.

### DNA isolation

For telomere-length assays, a-CGH, and methylation studies, genomic DNA of hBM-MSCs was extracted at different culture passages by using a Wizard Genomic DNA Purification Kit (Promega, Mannheim, Germany), according to the manufacturer's instructions. DNA concentration was determined on a NanoDrop ND-1000 spectrophotometer (NanoDrop Technologies, Berlin, Germany). In only three cases (P4 of donor 5, P0 of donor 6, and P4 of donor 8), the genomic DNA was amplified before array-CGH experiment, to enrich its amount, by using a GenomePlex Complete Whole Genome Amplification (WGA) kit (Sigma) according to the manufacturer's protocol. This kit allows an approximately 500-fold amplification of genomic DNA to be generated. In brief, 50 ng of genomic DNA was fragmented and converted to PCR-amplifiable OmniPlex Library molecules flanked by universal priming sites and then PCR-amplified by using universal primers for 14 cycles.

### hBM-MSCs telomere-length assay

To determine the hBM-MSCs telomere length, TeloTAGGG Telomere Length Assay (Roche Diagnostics, Mannheim, Germany) was used at different culture passages (P0, P3, P6, P9, and P12), according to the manufacturer's instructions. The positive control DNA supplied with the TeloTAGGG Telomere Length Assay is purified genomic DNA from immortal cell lines. A total of 1 to 1.5 μg genomic DNA was digested with an *Hinf I/Rsa I *mixture for 2 hours at 37°C. The sequence specificity of these two enzymes ensures that telomeric DNA and subtelomeric DNA is not cut, whereas nontelomeric DNA is digested to low-molecular-weight fragments. After DNA digestion, the DNA fragments were separated by 0.8% agarose gel electrophoresis and transferred, after being denaturated and neutralized, to a positively charged nylon membrane (Roche Diagnostics, Mannheim, Germany) by Southern blotting. The blotted DNA fragments were hybridized to a digoxigenin (DIG)-labeled probe specific for telomeric repeats at 42°C for 3 hours. The hybridized membrane was washed in a high-stringency buffer and incubated with a DIG-specific antibody covalently coupled to alkaline phosphatase (AP). After the final wash, AP substrate (CDP-Star), a highly sensitive chemiluminescent substrate, was applied and exposed on x-ray film for 10 to 20 minutes at 25°C. After exposure of the blot to x-ray film, the mean size of the different sample smears was compared with the molecular-weight marker.

### Array CGH Analysis

Genomic copy-number analysis was performed by using the Agilent Human Genome CGH Microarray 180 K kit (Agilent Technologies, Palo Alto, CA, USA) by following the manufacturer's recommendations, with a Genomic DNA (Female Promega, Mannheim, Germany) as reference. The analysis was performed by using Feature Extraction v10.7 and DNA Analytics v6.5 software (Agilent Technologies) applying the ADM2 algorithm with a threshold of 5, minimum absolute average log2 ratio in called intervals of 0.30, and a minimum of three probes. Putative chromosome copy-number changes were defined by intervals of three or more adjacent probes and were considered to be duplicated or deleted when results exceeded the ±0.30 range. All nucleotide positions were referred to the Human Reference Sequence (GRCh37) Assembly Feb. 2009 hg19 of UCSC.

### MeDIP-Chip

Methylated DNA immunoprecipitation and chip hybridization were performed by following the guidelines of Agilent Microarray Analysis of Methylated DNA Immunoprecipitation Protocol (Version 1.0, Agilent Technologies). Methylation analysis was performed on a genomic equimolar pool of DNA of hBM-MSCs from four different donors: donor 1 and donor 2 at P3; donor 5 and donor 6 at P6 (pool of early passages); and donor 1 at P9, donor 2 at P10, donor 5 at P12, and donor 6 at P9 (pool of late passages). The two pools were used in two independent experiments as reference samples (labeled with Cyanine 3); they were hybridized on a Human CpG island array (244 K format by Agilent Technologies), in competition with the respective methyl-DNA immunoprecipitated fractions (labeled with Cyanine 5). The array contained 237,220 probes (45- to 60-mer) representative of all 27,639 CpG islands in the human genome, at a density of about 1 probe per 100 bp. Data analysis was performed by using Genomic Workbench 6.5 and according to the model described by Straussman [27]. In brief, a value of Combined Z-score (*P *value) was assigned by Genomic Workbench 6.5 for each CpG island recognized by the probes on the array. For each experiment, a bimodal methylation curve was derived: the probe Z-scores for each island were averaged to obtain the Island Methylation Score (IMS) on the × axis, whereas the number of probes was on the Y axis. We then set numeric thresholds for determining the methylation status of each island. We calculated the distance between the demethylated (H1) and methylated (H2) peaks and set the upper and lower limits for DNA methylation as ±10% of this value from the IMS at the lowest point (L), located between the two peaks in the bimodal distribution curve. Islands with an IMS above the upper threshold were assigned a value of +1 (methylated), whereas islands with an IMS below the lower threshold were assigned a value of -1 (demethylated). Islands with an IMS between the two thresholds were considered undetermined (0).

### Gene ontology analysis

The gene ontology (GO) analysis was performed by using GOstat software [28] based on AmiGO (the Gene Ontology database, version 1.8) to identify possible enrichment of functional groups, related to "biologic process," in a specific input list of genes. The input list may contain genes delineated within gain and loss regions detected by a-CGH or genes resulted differentially methylated by MeDIP-CGI-array. GOstat software output file is a list of the *P *value for each GO term, estimating the probability that the observed counts could have occurred by chance. GO analysis was selected for the biologic processes, and a *P *< 0.05 was imposed. To limit the number of GO terms, a class should comprise more than five genes to be considered for further analysis [29]. In addition, GO terms were divided into seven functional categories: development and differentiation, metabolic process, cell cycle and growth, cell signaling, apoptosis and cell death, gene expression, and response to stimulus. Categories were ranked in order of the percentage of genes found, as described by Liu *et al. *[30]. The percentages of demethylated and methylated gene promoters were calculated for each category as follows: (total number of genes within a specific category)/(number of genes in the "input list" associated with a specific GO term). Because the same gene may belong to different GO processes, it was counted only once within a specific category. Conversely, because the same gene could belong to different categories of biologic processes, the sum of the percentages could not be 100%.

### Statistical analysis

Differences in telomere length among passages were analyzed by using one-way analysis of variance (ANOVA). For each donor and for each passage, a medium spot in the range of telomere length (smear) was calculated, and a mean value between donors was calculated for each passage. Data were expressed as mean ± SEM. Comparisons of mean values among the passages were analyzed by using a Tukey multiple-comparison test. A 5% probability (*P *< 0.05) was used as the level of significance.

To assess differences in CpG islands, methylation between the two pooled samples of hBM-MSC (donors 1, 2, 5, and 6), the percentages of each chromosome, "early" versus "late" passages, were compared by using the Student *t *test. Differences were considered statistically significant with *P *< 0.01.

A χ^2 ^test (*P *< 0.05) was performed to detect significant differences between GO-category percentages of demethylated versus methylated gene promoters, moving from early to late passages.

Prospective ethical approval was not sought; however, the Ethical Committee of the University of Milano-Bicocca analyzed the article retrospectively and recognized an overall correct development of the research, including the application of the standard informed-consent procedure in force at the San Gerardo Hospital.

## Results

### hBM-MSC Characterization

**Figure 1:**
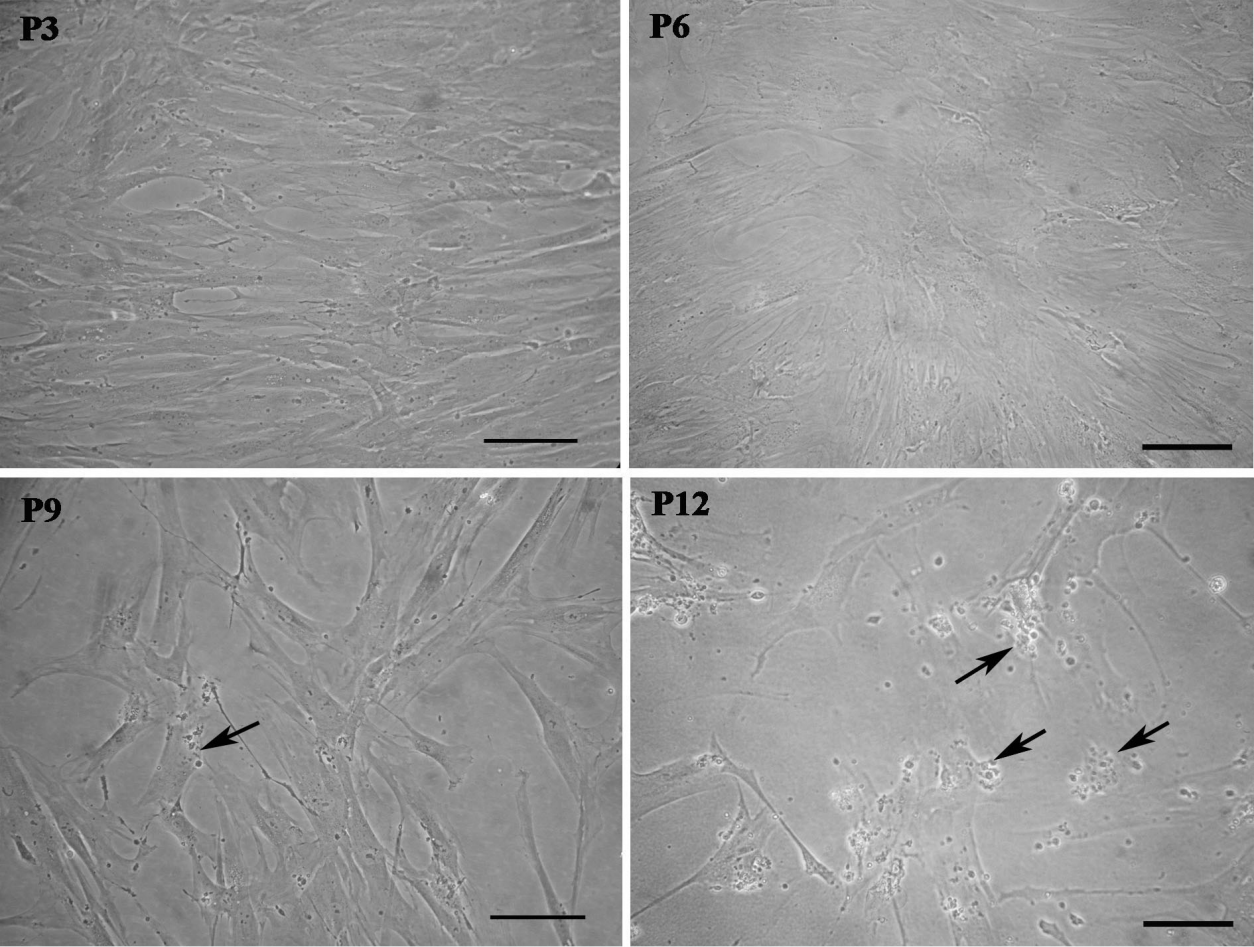
**Human bone marrow stem cell (hBM-MSC) cultures at P3, P6, P9, and P12**. At all culture passages examined, hBM-MSCs displayed a fibroblast-like morphology. At P9, some extra- and intracellular debris (arrows) appeared and became more evident at P12. Images from donor 6 are shown by way of example. Bars, 100 μm.

**Figure 2:**
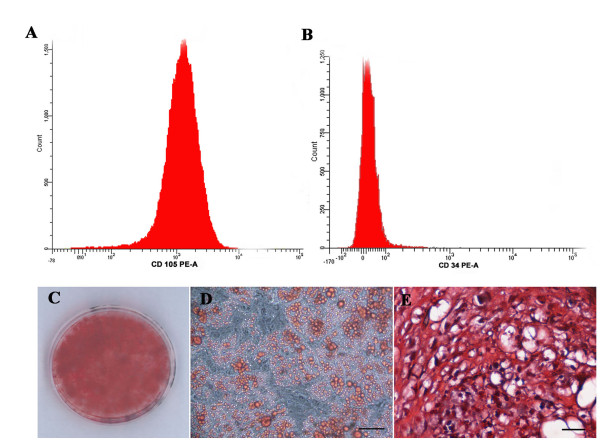
**Human bone marrow stem cells (hBM-MSCs) characterization**. hBM-MSC expression of CD105 **(A) **and CD34 **(B) **by flow-cytometric analysis. hBM-MSC mesengenic differentiation capability (C-E): Alizarin red staining of osteogenic differentiated hMSCs **(C)**; oil red O staining of adipogenic differentiated hBM-MSCs **(D)**; safranin O staining of chondrogenic differentiated hBM-MSCs **(E)**. Bars, 50 μm (D) and 25 μm (C).

**Figure 3:**
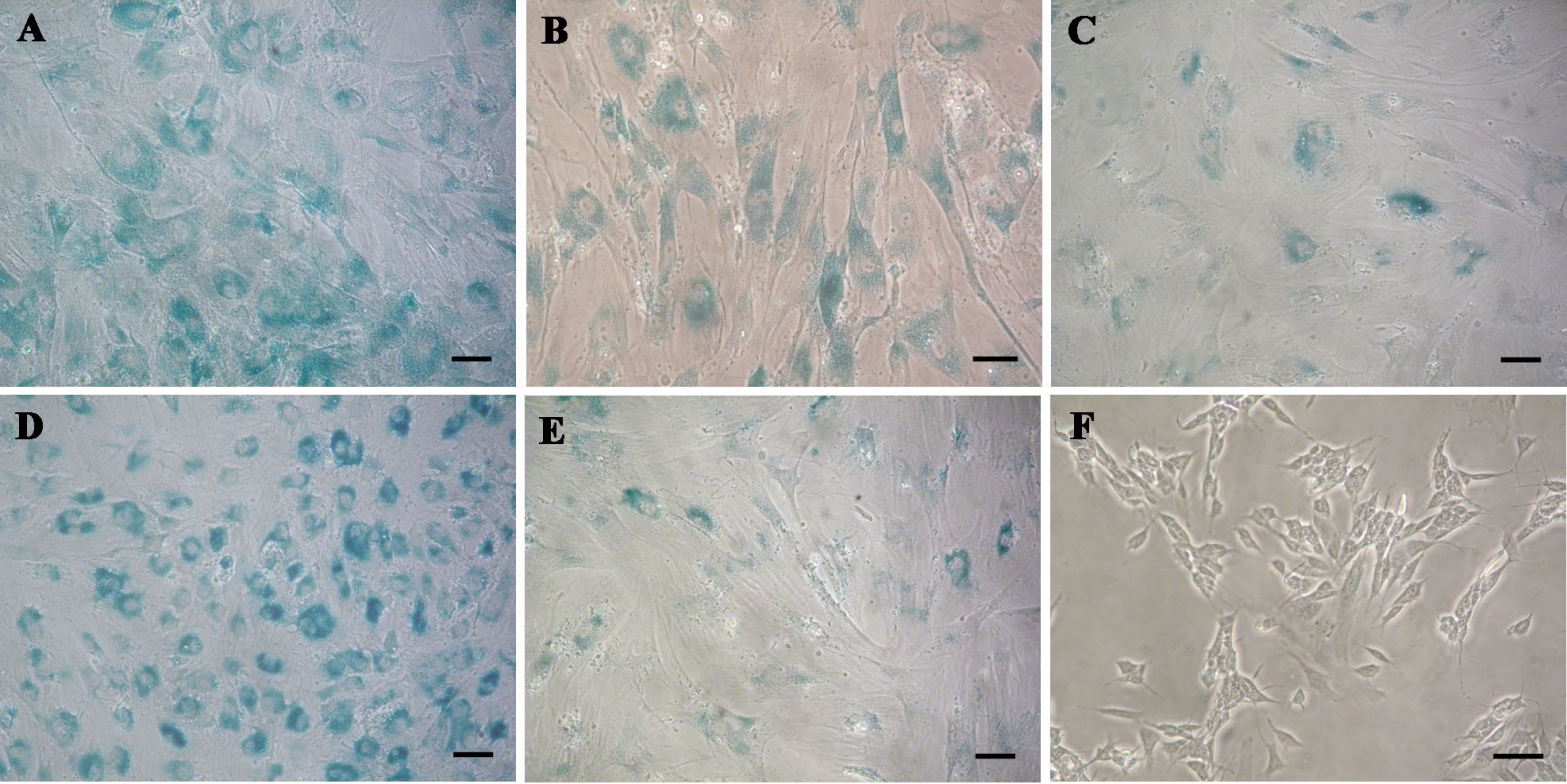
**Human bone marrow stem cell (hBM-MSC) senescence**. β-Galactosidase staining (blue) of hBM-MSCs from donor 2 at P10 **(A)**, donor 3 at P4 **(B)**, donor 5 at P13 **(C)**, donor 6 at P16 **(D)**, and donor 8 at P14 **(E)**. SHSY-5Y cell line **(F) **was used as negative control of the β-galactosidase staining. Bars, 50 μm.

The hBM-MSCs from eight anonymous healthy donors used in this study were characterized according to the criteria established by the International Society for Cellular Therapy [31]: they were plastic-adherent with a fibroblast-like morphology (Figure 1), positive for CD90, CD105 (Figure 2A), HLA-ABC, negative for CD33, CD34 (Figure 2B) and HLA-DR [23], and able to differentiate toward osteogenic, adipogenic, and chondrogenic lineages (Figure 2C through E). hBM-MSCs from several donors showed different proliferative capacities, and at the same culture passage, the population duplication time (PDT) varied greatly from one donor to another. For example, at P9, the PDT ranged from 24 hours (donor 5) to 97.75 hours (donor 1). Moreover, for hBM-MSCs from the same donor, the PDT increased with increasing passage (for example, for donor 2 at P6, it was 24 hours, and at P11, it was 68 hours). From P10 onward, for most of donors, cultures were characterized by the presence of abundant extra- and intracellular debris (Figure 1). The achievement of the senescence phase was variable among donors (Table 1), as demonstrated by β-galactosidase staining (Figure 3A through E). Negative control represented by SHSY-5Y neuroblastoma cell-line culture was characterized by β-galactosidase-negative cells (Figure 3F).

**Table 1:** Senescence β-galactosidase staining assay on hBM-MSCs from eight healthy donors

Donor	Passage	% Senescent cells (β-galactosidase^+^)
1	11	35
2	10	51.25
3	4	77.5
4	10	50
5	13	17.5
6	16	82.5
7	12	85
8	14	35

### hBM-MSCs chromosomal profile

The chromosomal stability of hBM-MSCs was evaluated with conventional cytogenetic analysis: at least three different passages were evaluated, except for donor 3, for whom data were available only for P3 (Table 2). All hBM-MSCs were generally characterized by a normal karyotype with a variable trend to random chromosome losses, most likely due to the technical preparation of chromosomes; conversely, random chromosome gains were much rarer. However, in two cases, the presence of clonal aneuploidies was evidenced. In one case (donor 4), at least 52% of metaphases at P9 presented trisomy of chromosome 7, also confirmed by fluorescence *in situ *hybridization (FISH) analysis (see Additional file 1, Figure S1); at P12, the same karyotype was found in 50% of the metaphases; moreover, in 11% of cells, a loss of one chromosome × occurred, so the total number of chromosomes was 46. In the second case (donor 5), two equally represented subpopulations were evidenced at P4: a normal one, and a second with karyotype: 49,XX,+5,+7,+9. However, further analysis at P6, P10, and P12 failed to reveal any clonal abnormalities, probably because of *in vitro *negative selection of the aneuploid clone.

**Table 2:** Conventional cytogenetic analysis on hBM-MSCs from eight healthy donors at several passages *in vitro*

**Donor**	**Passage**	**Norm. karyotype** **(%)**	**Clonal aneupl**. **(%)**	**Clonal struct**. **abnorm (%)**	**Random loss (%)**	**Random gain (%)**	**Metaphases (number)**	**Metaphases/Cells (number)**	**Mitotic index (%)**
**1** (46,XX)	7	45.5	0	0	54.5	0	22	24/1038	2.3
	9	66.7	0	0	33	0	39	8/1000	0.8
	12^a^	60	0	0	40	0	5	15/1000	1.5
									
**2** (46,XY)	6	82.8	0	0	15.6	1.6	64	70/1000	7.0
	10	80	0	0	20	0	30	24/1010	2.4
	16^a^	45.5	0	0	36.4	18.1	11	11/1007	1.1
									
**3** (46,XX)	3	70	0	0	26	4	23	4/1032	0.4
									
**4** (46,XX)	3	78	0	0	22	0	27	16/1000	1.6
	6	70.4	0	0	25.9	3.7	27	16/1000	1.6
	9	41.2	52.9 (47,XX,+7)	0	5.9	0	17	2/1320	0.2
	12	30.6	50 (47,XX,+7) and 11.1 (46,X,+7)	0	8.3	0	36	16/1000	1.6
									
**5** (46,XX)	4	33.3	33.3 (49,XX,+5,+7,+9)	0	33.3	0	18	12/1011	1.2
	6	80	0	0	12	8	25	7/1000	0.7
	10	81.3	0	0	18.7	0	32	14/1080	1.3
	12	92.3	0	0	7.7	0	26	18/1028	1.8
									
**6** (46,XY)	4	86	0	0	14	0	50	47/1000	4.7
	6	76.5	0	0	23.5	0	17	2/1001	0.2
	9	82.7	0	0	17.3	0	53	41/1000	4.1
	12^a^	72.7	0	0	18.2	9.1	11	1/1000	0.1
									
**7** (46,XX)	4	90.6	0	0	3.1	6.3	32	49/1011	4.8
	6	92.9	0	0	7.1	0	14	2/1000	0.2
	9	81.3	0	0	18.7	0	16	11/1029	1.1
									
**8** (46,XY)	4	100	0	0	0	0	37	27/1030	2.6
	6	78.1	0	0	17	4.9	41	22/1088	2.0
	9	77	0	0	23	0	30	6/1000	0.6

^a^In senescence. Clonal aneupl., clonal aneuploidies; Struct. abnorm., structural abnormalities.

### Telomere length in hBM-MSCs

**Figure 4:**
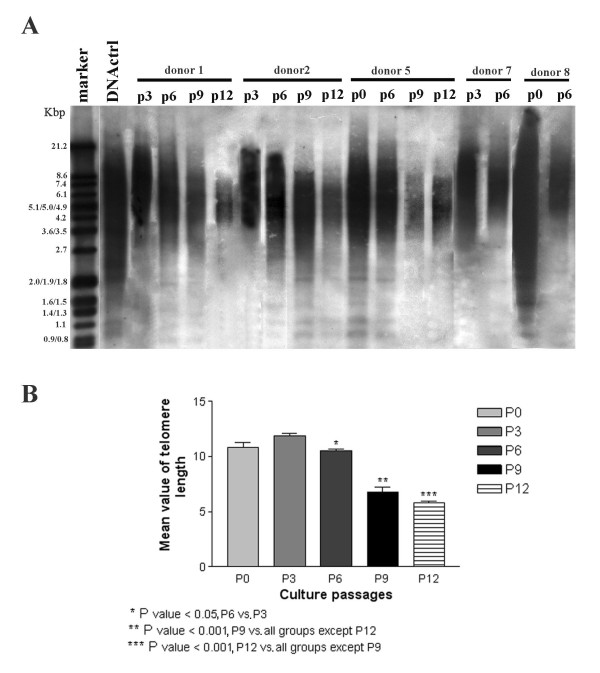
**Telomere length in human bone marrow stem cells (hBM-MSCs) at different culture passages**. **(A) **Telomere length evaluated by Southern blotting in hBM-MSCs (donors 1, 2, 5, 7, and 8) at P0, P3, P6, P9, and P12. The size marker is indicated on the left, and the positive control DNA used is a purified genomic DNA from immortal cell lines. Differences in telomere length were observed between P0, P3, and the following passages (P6, P9, P12) examined. **(B) **ANOVA statistical analysis of telomere length (donors 1, 2, 5, 7, and 8). A medium spot in the range of telomere length (smear, A) was calculated (see Material and Methods), and a mean value between donors (x axis) was calculated for each passage. Data are expressed as mean ± SEM. No significant differences in the mean values of telomere lengths were observed between P0 and P3, whereas significant differences were observed between P3 and P6 (*P *< 0.05), and between P0/P3/P6 and P9/P12 (*P *< 0.001).

Telomere length was assessed in hBM-MSCs from five donors at P0, P3, P6, P9, and P12. For two donors, telomere length was examined at P0: in one case (donor 5), it was comparable to the positive control, whereas in the other (donor 8), longer telomeres, not comparable to the positive control, were observed (Figure 4A). Regarding the next passages analyzed, no differences in telomere length were observed by comparing the same passage between different donors (Figure 4A). However, differences in telomere length were observed between early passages (P0, P3) and later ones (P6, P9, P12). As shown in Figure 4B, no significant differences in the mean value of telomere length were observed between P0 and P3, and between P9 and P12, whereas significant differences were observed between P3 and P6 (*P *< 0.05) and between the P0/P3/P6 group and the P9/P12 group (*P *< 0.001; ANOVA test).

### hBM-MSCs genomic profile

**Figure 5:**
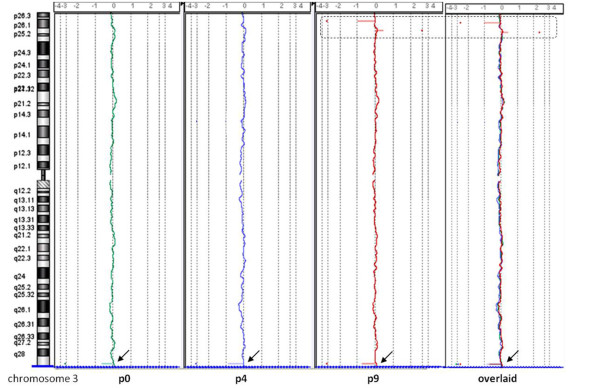
**Example of array-CGH profiles of human bone marrow stem cells (hBM-MSCs) at three different passages of culture**. Example of chromosome 3 from donor 8 in three overlapping experiments (P0, green line; P4, blue line; P9, red line): the profiles were almost overlapping (black arrow, a common copy-number variation (CNV), but P9 showed some exclusive CNVs (dotted rectangle).

We performed a detailed genomic study for hBM-MSCs derived from donors 5, 6, and 8 at P0, P4, and P9 and 10, for which it was possible to isolate an adequate number of cells. Molecular karyotyping was analyzed by means of Agilent Human Genome CGH Microarray 180 K kit (see Methods). The comparison of data from three experiments for each donor allowed us to discriminate between large copy-number variations (CNVs), constitutionally present in the donor's genome, and true chromosomal imbalances; Table S1 shows this in more detail (see Additional file 2). Array-CGH profiles of three experiments at different passages from the same donor were almost overlapping, and no significant deletions or duplications were demonstrated (Figure 5). The overall data showed that hBM-MSCs expanded *in vitro *confirmed a general stability of the genomic profile. We performed a GO annotation analysis to identify any possible enrichment of functional groups of genes within regions with gain and loss, but no statistically significant results were produced.

### hBM-MSCs epigenomic profile

**Figure 6:**
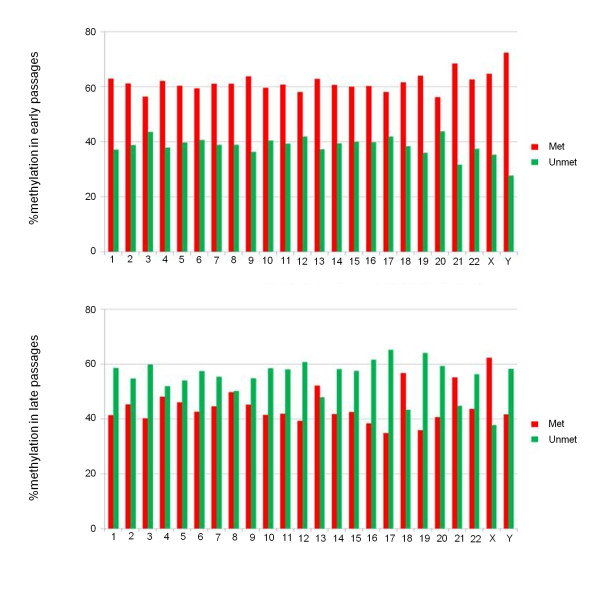
**CpG methylation profile of human bone marrow stem cells (hBM-MSCs) at early and late passages of culture**. Histograms with percentages of methylation of each chromosome at early passages (top) and late passages (bottom) of hBM-MSCs. Red, methylated; green, unmethylated.

To determine the effects of several passages in culture on DNA-methylation patterns, we analyzed the methylation profile of hBM-MSCs at early and late passages. We pooled hBM-MSC genomic DNA from four different donors (1, 2, 5, and 6) at early (P3 to P6) and late (P9, P10, and P12) passages, to average out any possible interdonor variation in methylation patterns and outline a kind of epigenomic signature specific for the two eras. Because we were interested in an overall profile of methylation and not in a specific genomic region, we applied the MeDIP-CGI-array (Agilent), a microarray platform that contains almost all of the CpG islands in the genome and that is a reliable solution for distinguishing highly methylated and unmethylated regions [32]. The distribution of CpG island methylation scores in both early and late passages shows a bimodal pattern, as previously described in a well-validated study [27]. We observed that, at early passages, 61.6% of all CpG islands were methylated, whereas 38.4% were unmethylated. Conversely, these percentages were reversed at late passages: 44.7% and 55.3%, respectively (see Figure S4 in Additional file 1). To assess differences in CpG islands, methylation between the two pooled hBM-MSC samples, "early" versus "late" passages, the percentages of each chromosome were compared by using Student *t *test (*P *< 0.01). Significant differences were observed in both cases. The reversal of methylation percentages between early and late passages was observed for all chromosomes, except for 18, 21, and X (Figure 6). Moreover, for chromosomes 4, 8, and 13, a reduction was noted in the difference between the two percentages.

**Figure 7:**
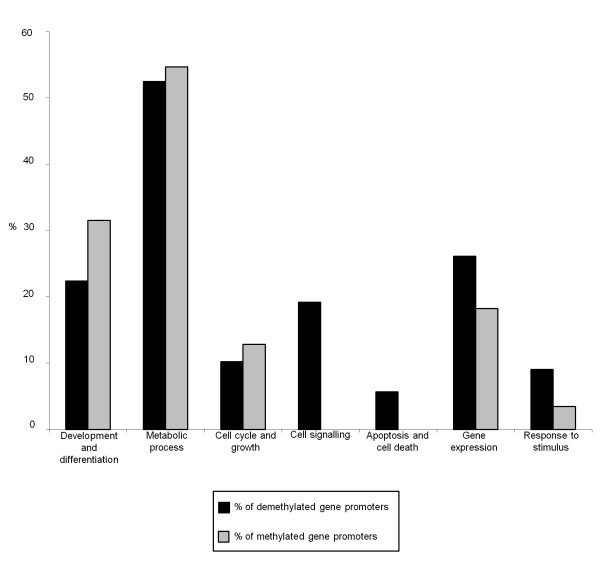
**Gene ontology (GO) analysis by GOstat software**. Histogram with percentages of gene promoters associated with a change in the methylation profile in late passages, classified by category of biologic process. Black, percentages of demethylated gene promoters; gray, percentages of methylated gene promoters.

To go beyond the identification of a series of individual genes with a changed methylation profile, we identified groups of functionally related genes, based on the GO system. Although it is well known that the methylation of DNA in 5 promoters suppresses gene expression, the role of DNA methylation in gene bodies is unclear [33]. For this reason, we decided to limit our analysis to only those genes with a change in their methylation status in CpG island promoters. In this way, the list of "demethylated gene promoters" (that is, changing from methylated to unmethylated) contains 1,284 genes; conversely, the list of methylated gene promoters (that is, changing from unmethylated to methylated) contains 518 genes. To simplify the interpretation of the large amount of data, we added the same criterion adopted by Aronica and colleagues [29] (that is, a class should comprise more than five genes to be considered for further analysis); in addition, we arbitrarily categorized the genes altered by extended passages into seven functional processes [30]. The Additional file 3 shows in more detail the two panels of GO terms, one specific for demethylated gene promoters and the other specific for methylated gene promoters (Additional file 3: Table S2A and B). The percentages of methylated gene promoters belonging to two categories, cell signaling and apoptosis and cell death, were found significantly different after extended passages (Figure 7) (χ^2 ^test; *P *< 0.05).

Finally, to achieve specific lists of GO terms (that is, exclusively present in the "demethylated gene promoters" or in the "methylated gene promoters" group), we eliminated all the redundant GO terms, common to these two sets (Table 3).

**Table 3:** Exclusive gene ontology terms of demethylated gene promoters at late passages of hBM-MSCs

Category	GO terms
Development and differentiation	GO:0007275 multicellular organismal development
	
	GO:0048468 cell differentiation/cell development
	
	GO:0007399 nervous system development
	
	GO:0032501 multicellular organismal process
	
	GO:0009887 multicellular organismal development/system development/organ development/organ morphogenesis
	
	GO:0030182 generation of neurons/neuron differentiation
	
	GO:0048699 generation of neurons
	
	GO:0022008 nervous system development/neurogenesis
	
	GO:0009790 multicellular organismal development/embryonic development
	
	GO:0048598 multicellular organismal development/embryonic development/embryonic morphogenesis
	
	GO:0048666 generation of neurons/neuron differentiation/neuron development
	
	GO:0009880 embryonic pattern specification
	
	GO:0048646 anatomic structure morphogenesis/anatomic structure formation
	
	GO:0000904 cell differentiation/cell development/cellular morphogenesis during differentiation
	
	GO:0031175 generation of neurons/neuron differentiation/neuron development/neurite development
	
	GO:0045597 positive regulation of cell differentiation
	
	GO:0048667 generation of neurons/neuron differentiation/neuron development/neuron morphogenesis during differentiation
	
	GO:0048812 generation of neurons/neuron differentiation/neuron development/neuron morphogenesis during differentiation/neurite morphogenesis
	
	GO:0007389 multicellular organismal development/pattern-specification process
	
	GO:0007417 nervous system development/central nervous system development
	
	GO:0045165 cell differentiation/cell-fate commitment

Metabolic process	GO:0016071 mRNA metabolic process
	
	GO:0009056 catabolic process
	
	GO:0044248 cellular catabolic process
	
	GO:0043285 biopolymer catabolic process
	
	GO:0006401 RNA catabolic process
	
	GO:0007005 mitochondrion organization and biogenesis
	
	GO:0009057 macromolecule catabolic process
	
	GO:0007584 response to nutrient
	
	GO:0016072 rRNA metabolic process
	
	GO:0031667 response to nutrient levels
	
	GO:0009310 amine catabolic process
	
	GO:0030163 protein catabolic process
	
	GO:0044270 nitrogen compound catabolic process
	
	GO:0044265 cellular macromolecule catabolic process
	
	GO:0006839 mitochondrial transport
	
	GO:0048878 chemical homeostasis
	
	GO:0009063 amino acid catabolic process
	
	GO:0045792 negative regulation of cell size

Cell cycle and growth	GO:0042127 regulation of cell proliferation
	
	GO:0045786 negative regulation of progression through cell cycle
	
	GO:0007088 regulation of mitosis
	
	GO:0000082 G_1_/S transition of mitotic cell cycle
	
	GO:0051329 interphase of mitotic cell cycle
	
	GO:0051325 interphase
	
	GO:0030308 negative regulation of cell growth

Cell signaling	GO:0007242 intracellular signaling cascade
	
	GO:0007169 transmembrane receptor protein tyrosine kinase signaling pathway
	
	GO:0048015 phosphoinositide-mediated signaling
	
	GO:0019932 second-messenger-mediated signaling
	
	GO:0045859 regulation of protein kinase activity
	
	GO:0043085 positive regulation of catalytic activity
	
	GO:0007167 enzyme-linked receptor protein signaling pathway
	
	GO:0007219 Notch signaling pathway
	
	GO:0045860 positive regulation of protein kinase activity
	
	GO:0033674 positive regulation of kinase activity
	
	GO:0007243 protein kinase cascade
	
	GO:0007200 G-protein signaling, coupled to IP3 second messenger (phospholipase C activating)
	
	GO:0000079 regulation of cyclin-dependent protein kinase activity
	
	GO:0007165 signal transduction
	
	GO:0043549 regulation of kinase activity

Apoptosis and cell death	GO:0008219 cell death
	
	GO:0016265 death
	
	GO:0006915 apoptosis
	
	GO:0043067 programmed cell death/regulation of programmed cell death
	
	GO:0043069 negative regulation of programmed cell death
	
	GO:0042981 regulation of apoptosis
	
	GO:0043066 negative regulation of apoptosis
	
	GO:0008632 apoptosis/apoptotic program
	
	GO:0006916 antiapoptosis
	
	GO:0043068 positive regulation of programmed cell death
	
	GO:0012501 programmed cell death
	
	GO:0012502 induction of programmed cell death

Gene expression	GO:0010467 gene expression
	
	GO:0006396 RNA processing
	
	GO:0006397 mRNA processing
	
	GO:0008380 RNA splicing
	
	GO:0006364 rRNA processing
	
	GO:0040029 regulation of gene expression, epigenetic
	
	GO:0000377 RNA splicing, via transesterification reactions with bulged adenosine as nucleophile
	
	GO:0000398 nuclear mRNA splicing, via spliceosome
	
	GO:0000375 RNA splicing, via transesterification reactions

Response to stimulus	GO:0042221 response to chemical stimulus
	
	GO:0006950 response to stress
	
	GO:0009605 response to external stimulus
	
	GO:0009991 response to extracellular stimulus;
	
	GO:0006955 immune response
	
	GO:0010035 response to inorganic substance
	
	GO:0009628 response to abiotic stimulus
	
	GO:0042060 wound healing

Exclusive GO terms of methylated gene promoters at LATE passages of hBM-MSCs	

Category	GO term

Development and differentiation	GO:0016043 cellular component organization and biogenesis
	
	GO:0008361 cell morphogenesis/regulation of cell size
	
	GO:0007276 gamete generation
	
	GO:0007283 spermatogenesis
	
	GO:0048232 male gamete generation

Metabolic process	GO:0006512 ubiquitin cycle
	
	GO:0031323 regulation of cellular metabolic process
	
	GO:0044255 cellular lipid metabolic process
	
	GO:0006629 lipid metabolic process
	
	GO:0006631 fatty acid metabolic process
	
	GO:0032787 monocarboxylic acid metabolic process
	
	GO:0043412 biopolymer modification
	
	GO:0006464 protein modification process
	
	GO:0043687 posttranslational protein modification
	
	GO:0008610 lipid biosynthetic process

Cell cycle and growth	GO:0000075 cell-cycle checkpoint
	
	GO:0016049 cell growth
	
	GO:0040008 growth/regulation of growth
	
	GO:0001558 regulation of cell growth

Response to stimulus	GO:0006974 response to DNA-damage stimulus
	
	GO:0006281 DNA repair

GO, gene ontology.

## Discussion

### The chromosomal-genomic profile of hBM-MSCs and the potential risk of malignant transformation

Stem cell-based therapy using hBM-MSCs holds promise for treating degenerative diseases, cancer, and repair of damaged tissues, for which currently no or limited therapeutic options exist. Despite the clinical potential of stem cell-based therapy, many risk factors were recently described as the "risk profile" by Herberts and colleagues [19]. Many identified risks derive from the requirement of *in vitro *expansion and/or differentiation of hBM-MSCs before administration to a patient. Cell-culture conditions may change the characteristics of BM-MSCs derived from human and from rat [7,34] because of intracellular and extracellular influences. In addition, every cell division has a small chance of introducing deleterious mutations, and mechanisms aimed at correcting these alterations may not function adequately during *in vitro *culture, eventually resulting in a tumorigenic phenotype. Several studies on MSCs from different sources have highlighted how genomic instability could lead to spontaneous immortalization and malignant transformation. Spontaneous malignant transformation of mouse BM-MSCs after long-term *in vitro *culture has been described [3-6]. Some publications have reported spontaneous transformation of hMSCs [11,12]. The same authors later retracted their data because of contamination with immortalized cell lines [13-15], but this topic is still open, and further studies are urgently needed to ensure the long-term safety of hMSCs.

The most important aspect of our study is that, for the first time to our knowledge, chromosomal, genomic, and epigenomic profiles of hBM-MSCs have been simultaneously evaluated and compared at different passages during *in vitro *propagation. In agreement with the data of Bernardo *et al. *[8], chromosomal stability of hBM-MSCs was evidenced for six of eight healthy donors; moreover, for two donors (4 and 5) clonal aneuploidies were found. In our study, as reported by others [35], the abnormal karyotype did not persist on prolonged culturing, probably because of clonal selection. Only trisomy of chromosome 7 in donor 4 seems to resist long periods of culture, also confirmed by FISH analysis (see Additional file 1, Figure S1). In addition, by study of chromosome heteromorphisms, we can rule out with sufficient certainty the possibility of contamination with other cell lines (see Additional file 1, Figure S2). The aneuploidy of human chromosome 7 might be especially interesting because we reported a functional trisomy, der(6;6), in our recent study on rat BM-MSCs [7], and several syntenic regions exist between these two chromosomes (see Additional file 1, Figure S3). One might speculate that some genes controlling the growth and division of cells in these syntenic regions may provide a selective advantage, and, indeed, changes in the number or structure of chromosome 7 occur frequently in human cancers. Even our array-CGH data confirmed a general stability of the genomic profile of hBM-MSCs. Culture-induced copy-number changes and loss of heterozygosity have been reported for human embryonal stem cell lines [35], but the clinical relevance with regard to tumorigenic potential of these genomic copy-number alterations still remains a matter of debate [36]. Moreover, stem cells may be considered potential candidates for malignant transformation, as some similarities exist between their features and those of cancer cells [36]. According to the cancer stem cell theory, only a small fraction of cells within a tumor (the cancer stem cells) are capable of tumor initiation, maintenance, and spreading [37]. However, despite the similarities between somatic stem cells and cancer stem cells, a direct link remains to be shown.

For many therapeutic applications, MSCs are used in an allogenic setting that might facilitate the efficient elimination of transformed cells by the immune system [38]. Therefore, the risk of hBM-MSCs would be restricted to autologous application in which the immune system is less efficient in eliminating the transformed cells [39].

### The epigenomic profile of hBM-MSCs and the replicative senescence process

The culture expansion of hBM-MSCs is limited, as it is for any other normal somatic cell. After a certain number of cell divisions, hBM-MSCs enter a senescent state and ultimately stop proliferating. This phenomenon, the Hayflick limit [16], is a continuous and organized process accompanied by far-reaching alterations in phenotype, differentiation potential, and global gene-expression patterns [21].

Several molecular mechanisms have been implicated in this phenomenon [40-42]. The progressive shortening of the telomeres has been proposed to be the main trigger for replicative senescence; because it functions "as an internal clock," with every cell division, the number of telomere repeats decreases. Progressive telomere shortening has also been demonstrated for MSCs [17,43]. Our data confirm these findings because no differences in telomere length were observed at early passages (P3 to P6) in hBM-MSCs from different donors. In contrast, at later passages (P9 to P12), telomeres were markedly shorter; furthermore, cell cultures were characterized by the presence of abundant extra- and intracellular debris and by a decreased proliferative capacity, as shown by the reduced PDT and the positivity to β-galactosidase staining, all clear signs of senescence. However, it is still being debated whether telomere shortening is really the initiating mechanism or whether it is instead effected by replicative senescence [44-46]. Telomere shortening may induce an antiproliferative signal resulting in cellular senescence, which, in turn, acts as a defense against cancer development.

As an alternative, it has been suggested that molecular switches (that is, epigenetic modifications) play a central role in regulating cellular aging [47,48]. Moreover, a direct link has been described between the maintenance of heterochromatic domains, such as those of centromeres and telomeres, chromosome-segregation defects, and abnormal telomere elongation [49]. Furthermore, a recent study evidenced that DNA methylation patterns were maintained throughout both long-term culture and aging, but highly significant differences were observed at specific CpG sites associated with promoter regions, especially in homeobox genes and genes involved in cell differentiation [22]. Then, the intimate correlation between DNA methylation-stem cell renewal-differentiation, as well as between stem cell culture-copy number changes-spontaneous malignant transformation is now evident (see reviews [19,20]).

In our study, we analyzed the DNA methylation levels of hBM-MSCs to delineate a kind of methylation signature specific for early and late passages. Because a great variability in terms of proliferative capacity and life span was evidenced between donors [8], with the aim of eliminating interindividual differences, we hybridized a pool of hBM-MSC genomic DNA from four different donors on a Human CpG island array (Agilent). We revealed a significant decrease in CpG island methylation levels of hBM-MSCs during long-term culture, in spite of early and late stages being quite close. Furthermore, a reversal of CpG island methylated and unmethylated percentages, between early and late passages, was observed for all chromosomes, except for 18, 21, and X. An explanation for these exceptions could be the lowest content of known protein-coding genes for 18 and 21; for chromosome X, it might be unbalanced, compared with other chromosomes, because 50% of donors in the pool were male. As it is clear that DNA methylation is necessary for controlling stem cell proliferation and differentiation [20], and a correlation between lineage-specific promoter hypermethylation and lack of differentiation capacity toward that lineage was found [50], further work is needed to investigate whether the changes we found on CpG island methylation levels of hBM-MSCs during long-term culture can affect their differentiation capabilities. Because it is commonly accepted that DNA methylation at CpG islands of gene promoters suppresses gene expression, we limited our GO analysis to genes with a change in the methylation status (from early to late passages) in CpG island promoters. Genes may have an antagonistic role in a specific biologic process, because some genes must be switched on, and others must be switched off, depending on whether they positively or negatively regulate this process. For this reason, the percentages of methylation and demethylation gene promoters could not significantly change for most of the biologic processes.

However, to limit the number of GO terms and to simplify the interpretation of the large amount of data, we applied the criteria of two published works [29,30]. Therefore, we considered GO terms that included more than five genes; these were arbitrarily divided into seven functional categories and were ranked in order of the percentage of genes found (see Additional file 3, Table S2A and B). A significant difference between the percentages of demethylated and methylated gene promoters was found only for two categories: "cell signaling" and "apoptosis and cell death"; indeed, these two groups were only in the "demethylated gene promoters in late passages" list (Figure 7 and Table S2A in Additional file 3). This means that these genes were unmethylated at early passages and remained so even at late passages. These two categories contain genes that have essential functions for the viability and functionality of MSCs (that is, the Notch signaling pathway, implicated in multiple cell-differentiation processes); thus, they should not be turned off.

After all, to achieve specific lists of GO terms (that is, exclusively present in the "demethylated gene promoters" or in the "methylated gene promoters" group), all the redundant GO terms common to these two sets were eliminated (Table 3).

For example, the "metabolic process" class in the "methylated gene promoters" group included several metabolic processes that could be inactivated with increasing passages. Among these, the majority were for lipid and fatty acid metabolic process (GO:0044255; GO:0006629; GO:0006631); interestingly, it was reported that adipogenic differentiation potential decreases during long-term culture [13-15]. Similarly, the GO:0008361 (cell morphogenesis-regulation of cell size) was an exclusive GO term included in the "development and differentiation" class of the methylated gene promoters group; this is quite interesting, because the majority of cells acquired a large and flat morphology at late passages.

Conversely, in the "demethylated gene promoters" group were listed genes that were methylated (that is, potentially turned off) at early passages and have become unmethylated (that is, potentially turned on) at late passages. Interestingly, numerous exclusive GO terms included in the "development and differentiation" class were involved in nervous system development, neurogenesis and neuron morphogenesis, and neuron differentiation (such as GO:0007399; GO:0030182; GO:0048699; and GO:0022008). This finding seems in agreement with the hypomethylation of the majority of the lineage-specific genes in MSCs reported by others [50]. Other potentially turned-on genes belong to the "gene expression" class; that is, regulation of gene expression and mRNA processing (such as GO:0010467; GO:0006396; and GO:0040029). Finally, the GO:0006955 "immune response" is the only one that has been highlighted as being underrepresented in the list of demethylated gene promoters. It would be interesting to investigate the significance of this observation because the immune-modulatory functions of MSCs could also change during culture expansion as a result of replicative senescence [21,38].

## Conclusions

In conclusion, our data indicate that long-term culture can affect several biologic features of MSCs. As a consequence, in a clinical setting, caution should be exerted before using hBM-MSCs for clinical applications. In addition, all the observed changes (that is, enlarged morphology, decreased number of cell divisions, random loss of genomic regions, telomere shortening) seem to belong to a definite program that is triggered and finely regulated by epigenetic modifications. This developmental process could lead to a reduction in the multipotent state of MSCs and might lead to tumor formation under specific conditions. It is very important to unravel further the epigenetic steps involved in this organized program during long-term culture of hBM-MSCs; thus appropriate tests should be applied to ensure the integrity of the genome and epigenome.

## Abbreviations

a-CGH: array comparative genomic hybridization; CNVs: copy number variations; der: derivative; GO: gene ontology; hBM-MSCs: human bone marrow mesenchymal stem cells; PDT: population-doubling time; QFQ: Q-bands by fluorescence using quinacrine.

## Competing interests

The authors declare that they have no competing interests.

## Authors' contributions

SR participated in the design of the study, performed the chromosomal-genomic and epigenomic profiles, data analysis and interpretation, and final approval of manuscript. AB participated in the design of the study, data analysis and interpretation, and manuscript writing. DF participated in the design of the study, carried out the experiments of hBM-MSCs characterization and telomere length, data analysis, and interpretation, helped to draft and gave final approval of the manuscript. MM participated in the design of the study, data analysis, and interpretation, helped to draft, and gave final approval of the manuscript. JR carried out the experiments of hBM-MSCs characterization and telomere length, data analysis and interpretation, and gave final approval of manuscript. GR carried out Gene Ontology data analysis and interpretation. SB carried out Gene Ontology data analysis and interpretation. LD conceived of the study and participated in its design and coordination, data analysis and interpretation, and final approval of the manuscript. GT conceived of the study, aided in data analysis and interpretation, and gave final approval of manuscript and financial support. All authors read and approved the final manuscript for publication.

## Supplementary Material

Additional file 1 **Figure S1-S4. Figure S1 **Left: Fluorescnce *in situ *hybridization (FISH) analysis with Vysis Williams Region FISH probe ELN (orange 7q11.23)/D7S486, D7S522 (green control probe) on P6 of Donor 4 confirmed the presence of two chromosomes 7. Right: FISH analysis with Poseidon EGFR, Her-1 (7p11; red) and SE7 (D7Z1; green control probe) on P9 of Donor 4 confirmed the presence of three signals for both probes (arrow) in about 50% of cells. **Figure S2 **Cytogenetic analysis at P6, P9, and P12 of Donor 4. For each passage, chromosome pairs of two different cells are aligned (from left to right: 3, 7, 13, and 21). Chromosome heteromorphisms (that is, normal variations in the appearance of chromosomes) of the centromere of chromosome 3, and of the short arms of chromosomes 13 and 21, exclude the presence of contamination with other cell lines. **Figure S3 **Syntenic regions between human chromosome 7 and rat chromosomes. The rat chromosome 6 is circled by the blue rectangle. From Ensemble Genome Browser [51]. **Figure S4 **CpG Methylation profile of human bone marrow mesenchymal stem cells (hBM-MSCs) at early and late passages of culture. Percentages of methylation of hBM-MSCs at early and late passages. Each symbol is associated with a different chromosome. The black horizontal lines indicate the average of the percentages of methylation. Met, methylated; Unmet, unmethylated. *P < 0.01. Click here for file

Additional file 2 **Table S1**. CNVs evidenced by array-CGH in human mesenchymal stem cells (hMSCs) at several passages in culture. The estimated percentage of mosaicism was calculated by using the formula determined by Cheung SW *et al. *[52]. Click here for file

Additional file 3 **Table S2**. GOstat analysis of demethylated and methylated gene promoters in late passages of human bone marrow mesenchymal stem cells (hBM-MSCs). Click here for file
