# Supplementary material for: From cytogenomic to epigenomic profiles: monitoring the biologic behavior of in vitro cultured human bone marrow mesenchymal stem cells
Source: Stem Cell Res Ther. 2012 Nov 20;3(6):47. doi: 10.1186/scrt138 (PMC3580477; doi:10.1186/scrt138)
Supplement: Additional file 1 — Figure S1-S4. Figure S1 Left: Fluorescnce in situ hybridization (FISH) analysis with Vysis Williams Region FISH probe ELN (orange 7q11.23)/D7S486, D7S522 (green control probe) on P6 of Donor 4 confirmed the presence of two chromosomes 7. Right: FISH analysis with Poseidon EGFR, Her-1 (7p11; red) and SE7 (D7Z1; green control probe) on P9 of Donor 4 confirmed the presence of three signals for both probes (arrow) in about 50% of cells. Figure S2 Cytogenetic analysis at P6, P9, and P12 of Donor 4. For each passage, chromosome pairs of two different cells are aligned (from left to right: 3, 7, 13, and 21). Chromosome heteromorphisms (that is, normal variations in the appearance of chromosomes) of the centromere of chromosome 3, and of the short arms of chromosomes 13 and 21, exclude the presence of contamination with other cell lines. Figure S3 Syntenic regions between human chromosome 7 and rat chromosomes. The rat chromosome 6 is circled by the blue rectangle. From Ensemble Genome Browser [51]. Figure S4 CpG Methylation profile of human bone marrow mesenchymal stem cells (hBM-MSCs) at early and late passages of culture. Percentages of methylation of hBM-MSCs at early and late passages. Each symbol is associated with a different chromosome. The black horizontal lines indicate the average of the percentages of methylation. Met, methylated; Unmet, unmethylated. *P < 0.01. [file scrt138-S1.PDF]

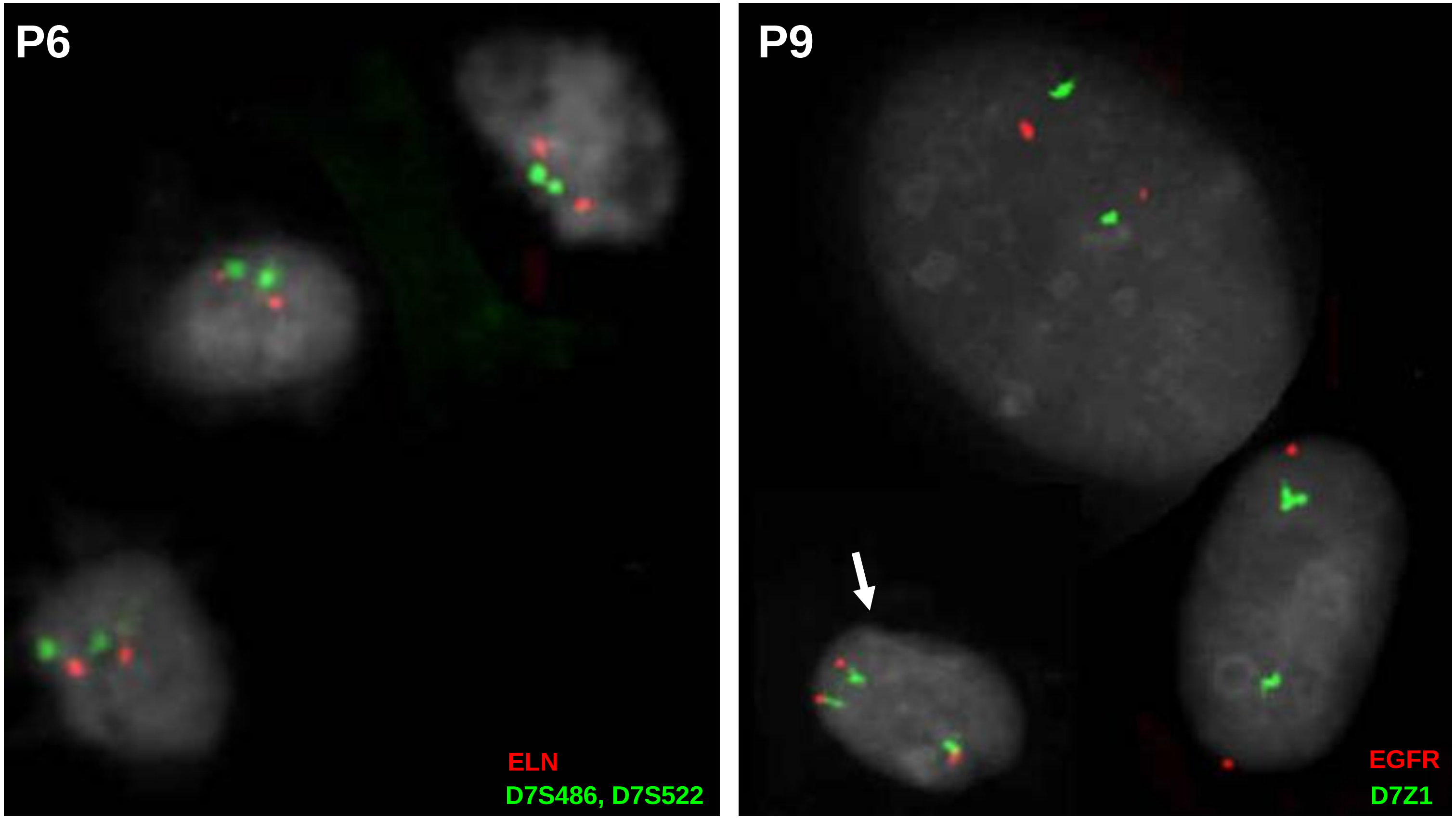

Figure S1.
Left: FISH analysis with Vysis Williams Region FISH probe ELN (orange 7q11.23)/D7S486, D7S522 (green control probe) on P6 of Donor 4 confirmed the presence of two chromosomes 7.
Right: FISH analysis with Poseidon EGFR, Her-1 (7p11; red) & SE7 (D7Z1; green control probe) on P9 of Donor 4 confirmed the presence of 3 signals for both probes (arrow) in about 50% of cells.

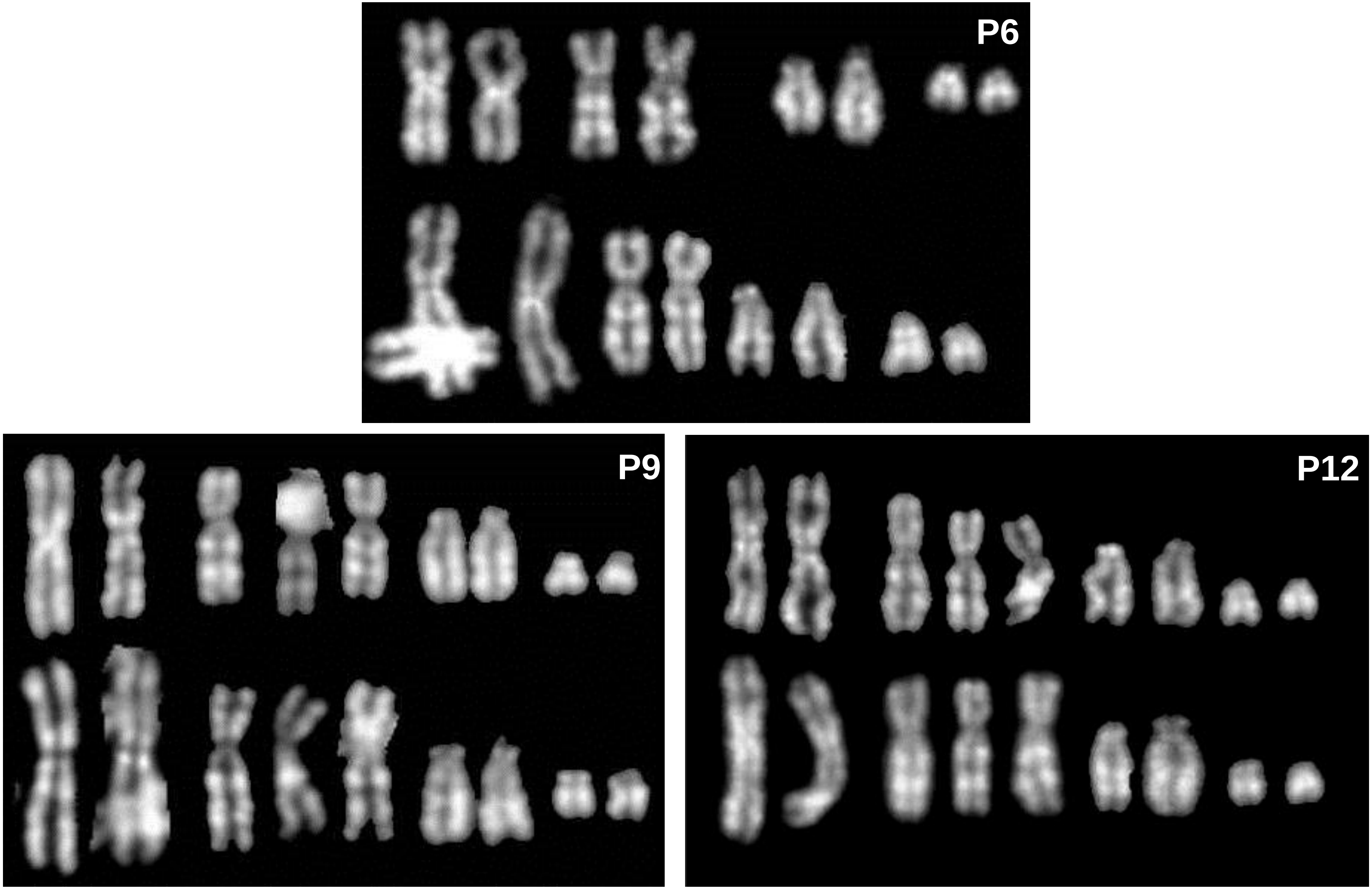

Figure S2.
Cytogenetic analysis at P6, P9 and P12 of Donor 4. For each passage chromosome pairs of two different cells are aligned (from left to right: 3, 7, 13 and 21). Chromosome heteromorphisms (i.e. normal variations in the appearance of chromosomes) of the centromere of chromosome 3, and of the short arms of chromosomes 13 and 21, exclude the presence of contamination with other cell lines.

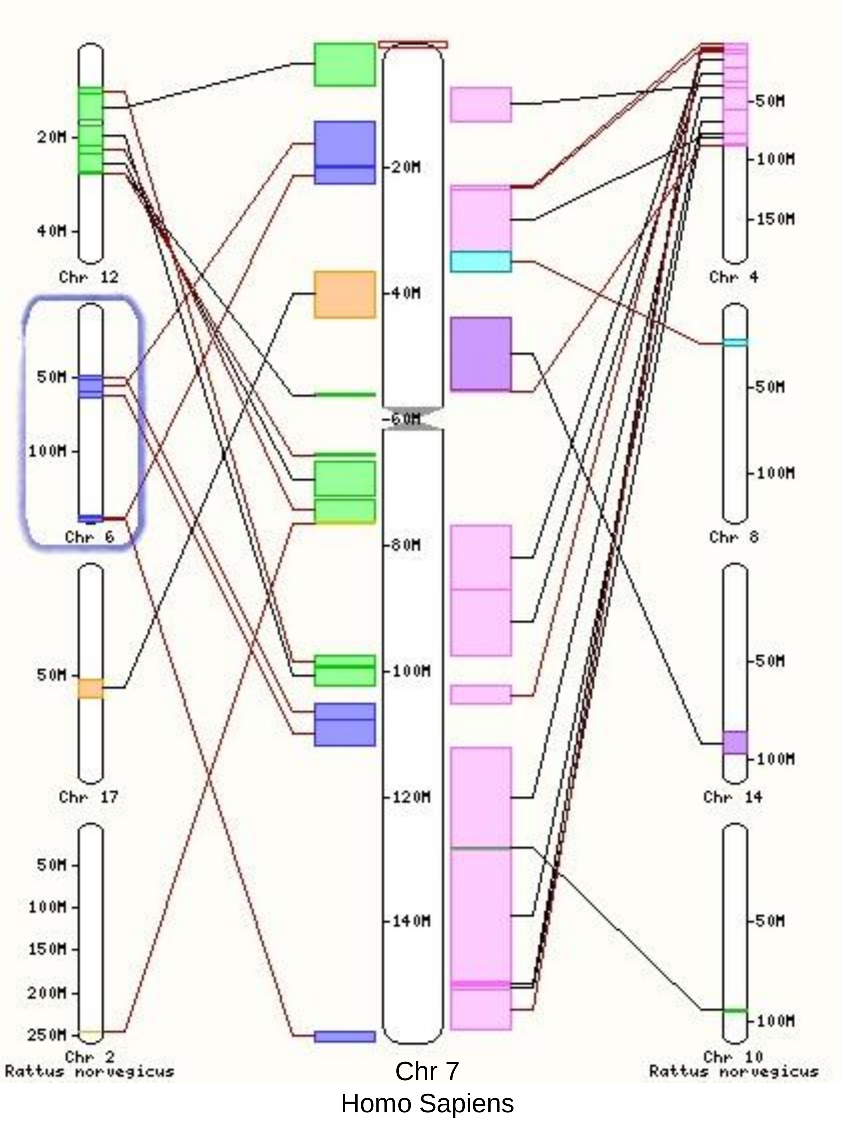

Figure S3.
Syntenic regions between human chromosome 7 and rat chromosomes. The rat chromosome 6 is circled by blue rectangle. From Ensembl Genome Browser [51].

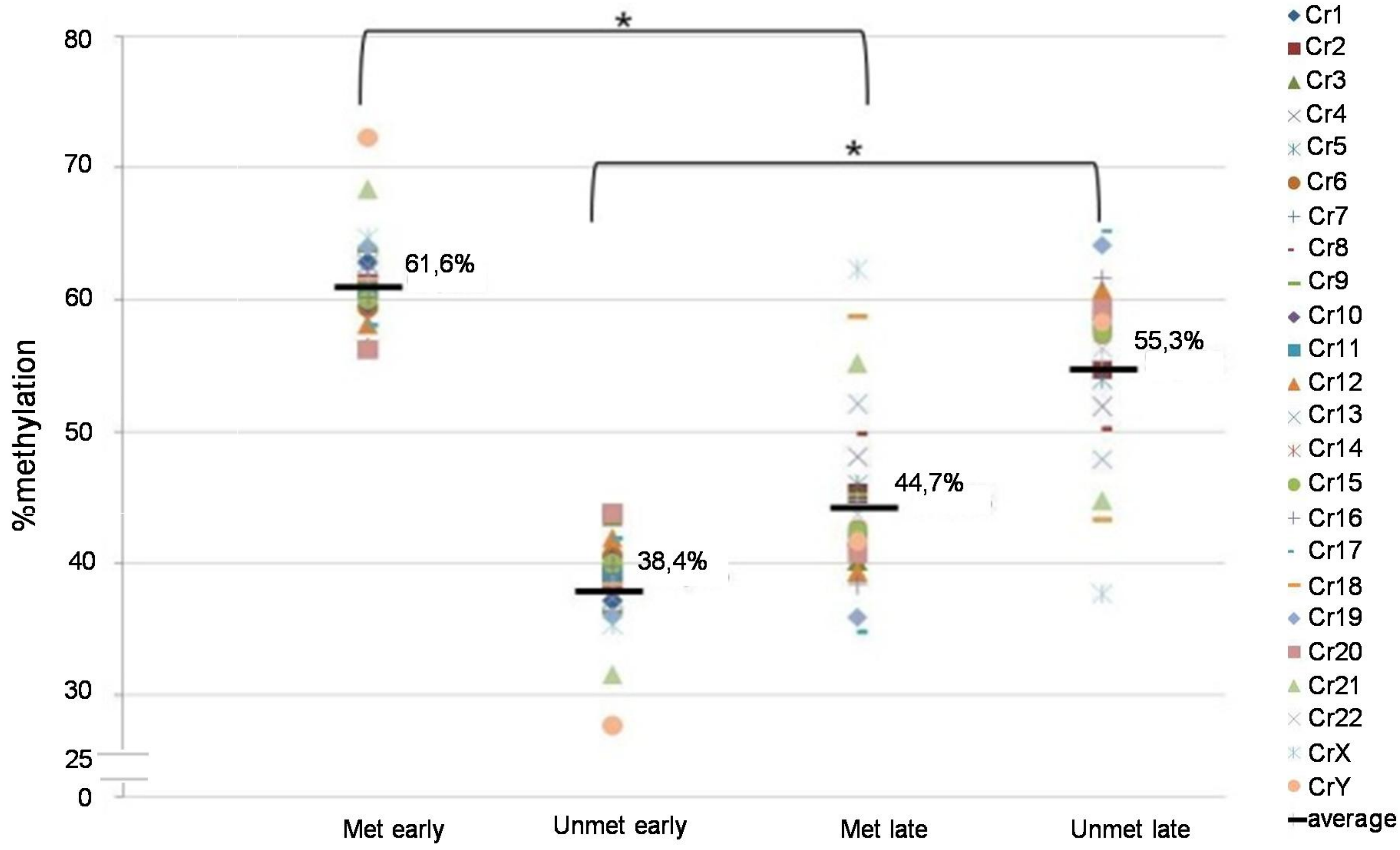

Figure S4.
CpG Methylation profile of hBM-MSCs at early and late passages of culture. Percentages of methylation of hBM-MSCs at early and late passages. Each symbol is associated with a different chromosome. The black horizontal lines indicate the average of the percentages of methylation. Met: methylated; Unmet: unmethylated. * *P* value<0.01
