# Supplementary material for: From cytogenomic to epigenomic profiles: monitoring the biologic behavior of in vitro cultured human bone marrow mesenchymal stem cells
Source: Stem Cell Res Ther. 2012 Nov 20;3(6):47. doi: 10.1186/scrt138 (PMC3580477; doi:10.1186/scrt138)
Supplement: Additional file 2 — Table S1. CNVs evidenced by array-CGH in human mesenchymal stem cells (hMSCs) at several passages in culture. The estimated percentage of mosaicism was calculated by using the formula determined by Cheung SW et al. [52]. [file scrt138-S2.DOC]

| **Table S1A. CNVs evidenced by array-CGH in hMSCs isolated from Donor 5.**  **Molecular karyotype: arr(1-22,X)x2** | | | |
| --- | --- | --- | --- |
| **CNV** | **P0** (*AMA log2ratio=0.19*) | **P4** (*AMA log2ratio=0.50***)** | **P10** (*AMA log2ratio=0.30***)** |
| **chr1**: 38102768-38142857; locus 1p34.3; 4 probes; 40.1Kb | - | - | Heterozygous loss |
| **chr1**: 38424446-38447630; locus 1p34.3; 3 probes; 23.3Kb | - | - | Heterozygous loss |
| **chr1**: 45242087-45269910; locus 1p34.1; 3 probes; 27.8Kb | Heterozygous loss 41% mosaic | Heterozygous loss 84% mosaic | Heterozygous loss |
| **chr1**: 47937232-47984700; locus 1p33; 5 probes; 27.8Kb | - | - | Homozygous loss |
| **chr2**: 31220458-31240698; locus 2p23.1; 3 probes; 30.2Kb | - | Amplification | - |
| **chr3**: 128678602-128706557; locus 3q21.3; 4 probes; 28Kb | Gain 66% mosaic | Amplification | - |
| **chr10**: 74040317-74095184; locus 10q22.1; 4 probes; 54.9Kb | Gain 79% mosaic | Amplification | - |
| **chr11**: 1880792-1901913; locus 11p15.5; 3 probes; 21.1Kb | - | - | Heterozygous loss |
| **chr11**: 57297500-57327540; locus 11q12.1; 4 probes; 30Kb | Gain 68% mosaic | Amplification | - |
| **chr11**: 64670780-64686471 [64686413-64701968]; locus 11q13.1; 3 probes; 15.7Kb | - | Heterozygous loss | [Heterozygous loss] |
| **chr12**: 105025071-105078814; locus 12q23.3; 4 probes; 53.7Kb | - | Amplification | - |
| **chr12**: 131733354-131815396 [131733354-131777299]; locus 12q24.33; 6[4] probes; 82Kb | Heterozygous loss 93% mosaic | [Heterozygous loss ; 84% mosaic] | Heterozygous loss |
| **chr14**: 21161962-21162335; locus 14q11.2; 3 probes; 0.37Kb | - | Amplification | - |
| **chr14**: 74001651-74022324; locus 14q24.3; 3 probes; 20.7Kb | Heterozygous loss | Heterozygous loss ; 81% mosaic | Heterozygous loss |
| **chr14**: 106531557-106559103; locus 14q32.33; 3 probes; 27.5Kb | Homozygous loss | Heterozygous loss | Heterozygous loss |
| **chr15**: 20432851-22558756; locus 15q11.1-q11.2; 55 probes; 2126Kb | Heterozygous loss ; 84% mosaic | Heterozygous loss | Heterozygous loss ; 85% mosaic |
| **chr15**: 25425317-25491412; locus 15q11.2; 7 probes; 66.1Kb | - | - | Heterozygous loss ; 85% mosaic |
| **chr15**: 76972308-77121841; locus 15q24.3; 20 probes; 149.5Kb | - | Heterozygous loss ; 80% mosaic | - |
| **chr16**: 2145917-2216380; locus 16p13.3; 5 probes; 70.5Kb | Gain | Amplification | - |
| **chr16**: 89653086-89888566; locus 16q24.3; 22 probes; 235.5Kb | Gain; 83% mosaic | Gain; non mosaic | - |
| **chr17**: 21195549-21327524; locus 17p11.2; 12 probes; 131Kb | - | - | Heterozygous loss ; 70% mosaic |
| **chr17**: 37323655-37377720; locus 17q12; 6 probes; 54.1Kb | Gain; 71% mosaic | Amplification | - |
| **chr17**: 41965843-42020245; locus 17q21.31; 5 probes; 54.4Kb | Gain; 69% mosaic | Amplification | - |
| **chr17**: 72363726-72390582; locus 17q25.1; 3 probes; 26.9Kb | Gain; 79% mosaic | Amplification | - |
| **chr19**: 5801521-5832928; locus 19p13.3; 4 probes; 31.4Kb | - | - | Heterozygous loss |
| **chr19**: 45852503-45884858; locus 19q13.32; 3 probes; 32.4Kb | Gain | Amplification | Gain |
| **chr19**: 54617703-54660834; locus 19q13.42; 5 probes; 43.1Kb | - | - | Heterozygous loss |
| **chr20**: 23741115-23835530; locus 20p11.21; 5 probes; 94.4Kb | - | - | Heterozygous loss ; 91% mosaic |
| **chr22**: 20754422-20777087; locus 22q11.21; 3 probes; 22.7Kb | Gain | Amplification | - |
| **chr22**: 24040187-24075717; locus 22q11.23; 4 probes; 35.5Kb | Gain | Amplification | - |
| **chr22**: 50613880-50649428; locus 22q13.33; 6 probes; 35.5Kb | - | - | Heterozygous loss ; 87% mosaic |
| **chrX**: 19832021-19923342; locus Xp22.12; 10 probes; 91.3Kb | - | Gain | - |
| **chrX**: 155097155-155223860; locus Xq28; 25 probes; 126.7Kb | - | - | Heterozygous loss ; 69% mosaic |

| **Table S1B. CNVs evidenced by array-CGH in hMSCs isolated from Donor 6. Molecular karyotype: arr(1-22)x2,(XY)x1** | | | |
| --- | --- | --- | --- |
| **CNV** | **P0** (*AMA log2ratio=0.50*) | **P4** (*AMA log2ratio=0.35*) | **P9** (*AMA log2ratio =0.35*) |
| **chr1**: 43909652-43919633; locus 1p34.2; 3 probes; 10Kb | - | Amplification | Amplification |
| **chr1**: 149852674-149879361; locus 1q21.1; 7 probes; 26.7Kb | - | - | Gain |
| **chr3**: 123352298-123367869; locus 3q21.1; 3 probes; 15.6Kb | Amplification | - | - |
| **chr3**: 184659811-184709108; locus 3q27.2; 6 probes; 49.3Kb | - | Heterozygous loss ; 59% mosaic | Heterozygous loss ; 84% mosaic |
| **chr3**: 195804728-195941396; locus 3q29; 8 probes; 136.7Kb | Gain | Gain; 95% mosaic | Gain; 77% mosaic |
| **chr4**: 7643994-7683855; locus 4p16.1; 4 probes; 39.9Kb | Amplification | Gain | Gain; 83% mosaic |
| **chr4**: 8155010-8195069; locus 4p16.1; 3 probes; 40.1Kb | Amplification | Gain; 62% mosaic | Gain; 66% mosaic |
| **chr4**: 108949257-108969448; locus 4q25; 3 probes; 20.2Kb | Amplification | - | - |
| **chr4**: 113596585-113733369; locus 4q25; 9 probes; 136.8Kb | Gain | - | - |
| **chr4**: 165955690-166015583; locus 4q32.3; 5 probes; 59.9Kb | Amplification | - | - |
| **chr5**: 58019626-58038333; locus 5q11.2; 3 probes; 18.7Kb | - | Heterozygous loss ; 77% mosaic | Heterozygous loss |
| **chr5**: 157342124-157394869; locus 5q33.3; 5 probes; 52.7Kb | Amplification | - | - |
| **chr7**: 1030366-1081240; locus 7p22.3; 8 probes; 50.9Kb | - | Amplification | Amplification |
| **chr7**: 8145326-8170225; locus 7p21.3; 4 probes; 25Kb | Amplification | - | - |
| **chr7**: 152913547-153022042; locus 7q36.2; 3 probes; 108.5Kb | Amplification | - | - |
| **chr7**: 154645405-154722505 [154696057-154722505]; locus 7q36.2; 8[3] probes; 77.1[24.4]Kb | Amplification | [Gain] | [Gain] |
| **chr8**: 120976905-121021524; locus 8q24.12; 4 probes; 44.6Kb | Amplification | - | - |
| **chr8**: 145959136-146027569; locus 8q24.3; 6 probes; 68.4Kb | Amplification | - | - |
| **chr9**: 95441416-95461353; locus 9q22.31; 3 probes; 20Kb | Amplification | - | - |
| **chr10**: 5965561-6010159; locus 10p15.1; 5 probes; 44.6Kb | Amplification | - | - |
| **chr10**: 22540230-22639751; locus 10p12.31-p12.2; 9 probes; 99.5Kb | Heterozygous loss ; 90% mosaic | - | - |
| **chr10**: 43755511-43868356; locus 10q11.21; 10 probes; 112.8Kb | - | - | Gain |
| **chr11**: 3023174-3091469; locus 11p15.4; 6 probes; 68.3Kb | Amplification | - | - |
| **chr11**: 55377910-55450788; locus 11q11; 8 probes; 72.9Kb | - | Gain | Gain |
| **chr11**: 62213579-62245885; locus 11q12.3; 3 probes; 32.3Kb | Amplification | Gain | - |
| **chr11**: 68229809-68287033; locus 11q13.2; 5 probes; 57.2Kb | Heterozygous loss ; 76% mosaic | - | - |
| **chr12**: 2924743-2959765; locus 12p13.33; 3 probes; 35Kb | Amplification | - | - |
| **chr12**: 8098442-8145192; locus 12p13.31; 4 probes; 46.8Kb | Amplification | - | - |
| **chr12**: 9637323-9713425; locus 12p13.31; 7 probes; 76.1Kb | Heterozygous loss | Homozygous loss | Homozygous loss |
| **chr12**: 121017665-121068023; locus 12q24.31; 5 probes; 50.4Kb | Amplification | Gain; 62% mosaic | Gain |
| **chr13**: 114756824-114773099; locus 13q34; 3 probes; 16.3Kb | - | Amplification | Amplification |
| **chr14**: 20217288-20427242; locus 14q11.2; 12 probes; 210Kb | - | Heterozygous loss ; 61% mosaic | Heterozygous loss ; 70% mosaic |
| **chr14**: 75116525-75139542; locus 14q24.3; 3 probes; 23Kb | Amplification | - | - |
| **chr14**: 104061473-104097810; locus 14q32.33; 4 probes; 36.3Kb | Amplification | - | - |
| **chr16**: 2776772-2823495; locus 16p13.3; 5 probes; 46.7Kb | Amplification | - | - |
| **chr16**: 3511539-3536965; locus 16p13.3; 3 probes; 25.4Kb | Amplification | Gain; 62% mosaic | Gain; 69% mosaic |
| **chr16**: 15816284-15849363; locus 16p13.11; 4 probes; 33.1Kb | Amplification | Gain; 89% mosaic | Gain; 85% mosaic |
| **chr16**: 58694880-58712078; locus 16q21; 3 probes; 17.2Kb | Amplification | Gain; 71% mosaic | Gain |
| **chr17**: 16822683-16853363; locus 17p11.2; 3 probes; 30.7Kb | Amplification | Gain | Gain |
| **chr17**: 40318960-40336980; locus 17q21.2; 4 probes; 18Kb | - | Amplification | Amplification |
| **chr18**: 57498468-57529162; locus 18q21.32; 3 probes; 30.7Kb | Amplification | - | - |
| **chr19**: 1439151-1462368; locus 19p13.3; 3 probes; 23.2Kb | Gain | Amplification | Amplification |
| **chr20**: 31456186-31489311; locus 20q11.21; 4 probes; 33.1Kb | Amplification | - | - |
| **chr20**: 49228692-49237741; locus 20q11.13; 3 probes; 9.1Kb | Amplification | Gain | Gain |
| **chr20**: 62087796-62163473; locus 20q13.33; 5 probes; 75.7Kb | - | Amplification | Gain |
| **chr21**: 18882379-18917591; locus 21q21.1; 3 probes; 35.2Kb | Amplification | - | - |
| **chr21**: 33954610-34001084; locus 21q22.1; 4 probes; 46.5Kb | Amplification | - | - |
| **chr22**: 27205669-27234452; locus 22q12.1; 3 probes; 28.8Kb | Amplification | - | - |
| **Table S1C. CNVs evidenced by array-CGH in hMSCs isolated from Donor 8. Molecular karyotype: arr(1-22)x2,(XY)x1** | | | |
| **CNV** | **P0** (*AMA log2ratio=0.20***)** | **P4** (*AMA log2ratio=0.38***)** | **P9** (*AMA log2ratio=0.32***)** |
| **chr2**: 80272577-80356041; locus 2p12; 6 probes; 83.5Kb | Heterozygous loss ; 77% mosaic | Heterozygous loss ; 66% mosaic | Heterozygous loss ; 59% mosaic |
| **chr3**: 4637891-4665790; locus 3p26.1; 3 probes; 27.9Kb | - | - | Heterozygous loss |
| **chr3**: 10061464-10203614; locus 3p25.3; 15 probes; 140.2Kb | - | - | Gain; 77% mosaic |
| **chr3**: 195419168-195427855; locus 3q29; 9 probes; 8.7Kb | Heterozygous loss ; 71% mosaic | Heterozygous loss ; 89% mosaic | Heterozygous loss ; 71% mosaic |
| **chr6**: 307939-378956; locus 6p25.3; 7 probes; 71Kb | Gain; 83% mosaic | - | - |
| **chr6**: 162145577-162170592; locus 6q26; 3 probes; 25Kb | - | - | Heterozygous loss |
| **chr6**: 163265954-163340236; locus 6q26; 7 probes; 74.3Kb | - | - | Heterozygous loss ; 79% mosaic |
| **chr7**: 1064151-1081240; locus 7p22.3; 3 probes; 17.1Kb | Amplification | Gain | Amplification |
| **chr8**: 39249352-39345479; locus 8p11.22; 9 probes; 96.1Kb | Gain | - | - |
| **chr10**: 4492966-4550976; locus 10p15.1; 4 probes; 58Kb | - | Heterozygous loss | - |
| **chr14**: 20203449-20414232; locus 14q11.2; 12 probes; 210.8Kb | Heterozygous loss ; 82% mosaic | Heterozygous loss ; 72% mosaic | Heterozygous loss ; 71% mosaic |
| **chr15**: 20432851-22409391; locus 15q11.1-q11.2; 40 probes; 1977Kb | Heterozygous loss ; 69% mosaic | Heterozygous loss ; 73% mosaic | Heterozygous loss ; 66% mosaic |
| **chr16**: 32573808-33625989; locus 16p11.2; 18 probes; 1052Kb | Heterozygous loss ; 49% mosaic | - | - |

AMA: Absolute Minimum Average

CNV: Copy Number Variation

Amplification: log2ratio > +1

Homozygous loss: log2ratio < -1.70

% of mosaicism: =|(2^(log2ratio)-1)/0.5|x100 [52].
